# Case Report: Adult degenerative scoliosis in two patients treated with percutaneous spinal endoscopic-assisted lumbar interbody fusion and percutaneous pedicle screw fixation

**DOI:** 10.3389/fsurg.2022.730504

**Published:** 2023-01-06

**Authors:** Jian-wei Du, Lei-ming Zhang, Yu-qiu Yan, Ya-ning Zhang, Xue-qin Rong, Song-hua Xiao, Xi-feng Zhang

**Affiliations:** ^1^ Department of Orthopedics, Affiliated Hospital of Yangzhou University, Yangzhou, China; ^2^ Department of Neurosurgery, The Sixth Medical Center of PLA General Hospital, Beijing, China; ^3^ Department of Spine Surgery, Beijing Aiyuhua Hospital, Beijing, China; ^4^ Department of Spinal Surgery, Linfen People’s Hospital, Linfen, China; ^5^ Department of Pain Treatment, The Third people’s Hospital of Hainan Province, Sanya, China; ^6^ Department of Orthopedics, Tsinghua University Affiliated Beijing Tsinghua Changgung Hospital, Beijing, China

**Keywords:** endoscopy, minimally invasive surgery, adult degenerative scoliosis, Cobb angles 3, orthopedics

## Abstract

Adult degenerative scoliosis (ADS) is a serious disease that often affects middle-aged and elderly people. ADS does not only cause sagittal and coronal deformity of the lumbar spine but also causes severe back and leg pain secondary to the compression of the neural structures. Open surgery remains the main method for correcting the occurring deformity and decompression of the neural structures; however, its benefit is limited in cases of large trauma. Minimally invasive spinal (MIS) surgery is an alternative method that has recently witnessed rapid development. It has the advantage of providing rapid recovery with less trauma as compared to conventional open surgery. We report two cases of ADS treated with percutaneous spinal endoscopic-assisted lumbar interbody fusion (EALIF) and percutaneous pedicle screw fixation. Both cases had moderate deformities of the lumbar spine (load-sharing classification 4–7 points) with severe back and leg pain, and they underwent successful MIS surgery. At 6 months of follow-up, the visual analog scale and Oswestry disability index scores of both patients improved and the deformity was corrected. For moderate ADS, percutaneous spinal EALIF and percutaneous pedicle screw fixation may achieve an effective correction of the deformity with direct decompression of neural structures.

## Introduction

Adult degenerative scoliosis (ADS) is a morphological and functional abnormality of the spine resulting from asymmetric degenerative changes in facet joints and intervertebral discs, aging, osteoporosis, and compression fractures (1–3). Currently, open spinal surgery remains the conventional method to treat ADS (4). However, there are many downsides to this technique, such as the large surgical range, the prolonged time of surgery, and the amount of resulting trauma. Moreover, middle-aged and elderly patients with comorbid conditions have a greater risk of developing complications.

With the continued progress of surgical techniques, microinvasive spinal (MIS) surgical techniques have recently witnessed rapid development. Compared with traditional open surgery, MIS has the advantage of providing rapid recovery with less trauma, and its indication can be effectively conducted by the minimally invasive spinal deformity surgery (MISDEF2) algorithm (5). Herein, we report two cases of ADS undergoing percutaneous lumbar interfusion and percutaneous pedicle screw fixation with the assistance of a spinal endoscope.

## Case description

### Case 1

**Figure 1:**
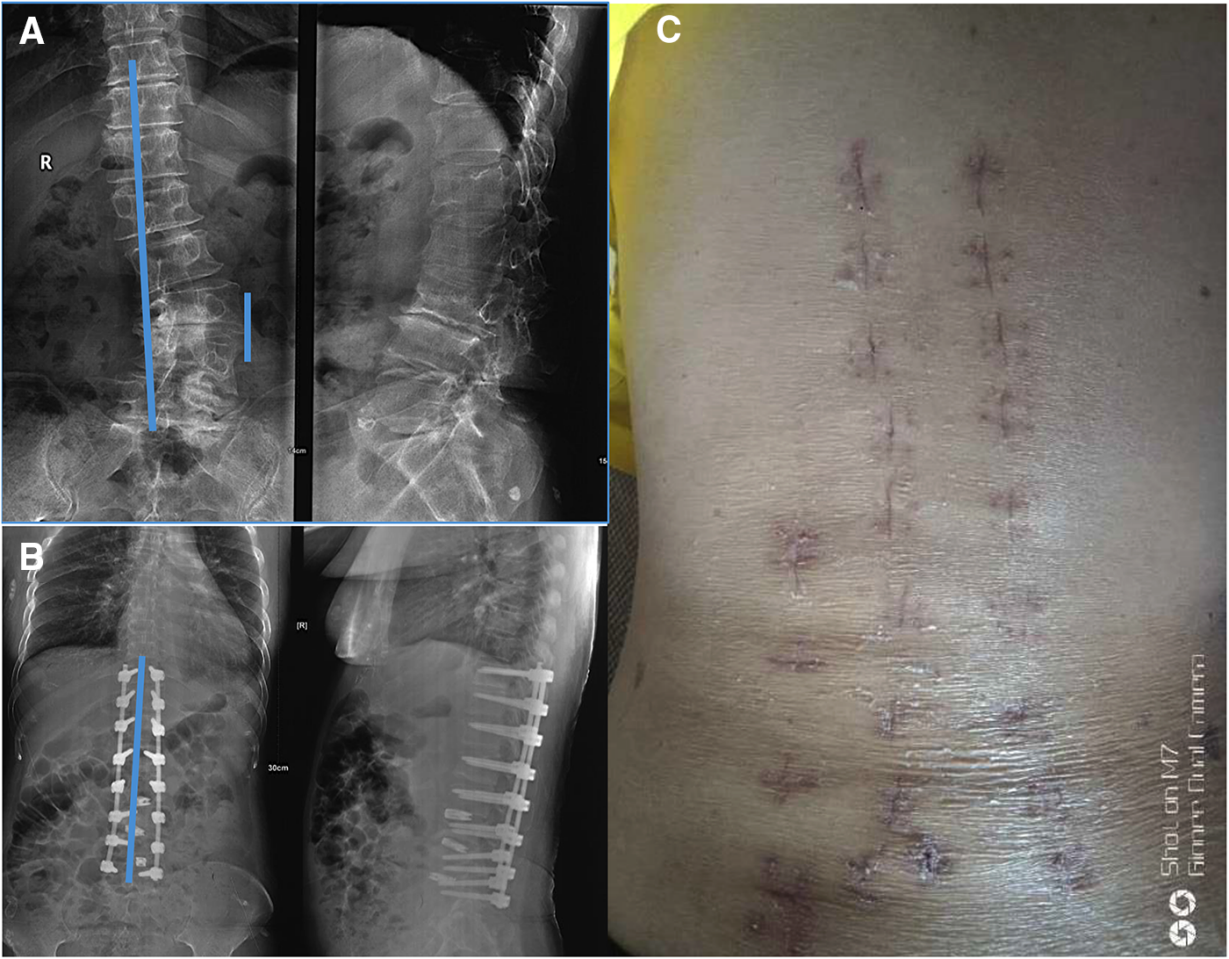
X-rays of the 56-year-old female patient with ADS, which was taken on November 2018: (**A**) before operation, (**B**) after operation, and (**C**) the patient's postoperative appearance.

A 56-year-old female patient complained of back pain for 20 years and left lower limb pain for 7 years that aggravated for 20 days. There were no cardiopulmonary deficits and no other underlying diseases. The muscle power, including the bending of hip and knee, extending the knee, dorsum at hallux and metatarsal flexion at hallux of both lower limbs was level 5. Accordingly, a diagnosis of ADS was established. At baseline, the visual analog scale (VAS) was 9 for back pain and 6 for leg pain, and the Oswestry disability index (ODI) score was 76%. Moreover, the Silva–Lenke type was IV. Radiological examinations showed lumbar scoliosis with a Cobb angle of 30 and lumbar kyphosis (LK) with an angle of 16 (Figure 1). In the past 2 years, the pain was severe, and it was managed with traditional Chinese acupuncture and massage. However, the condition did not improve.

### Case 2

**Figure 2:**
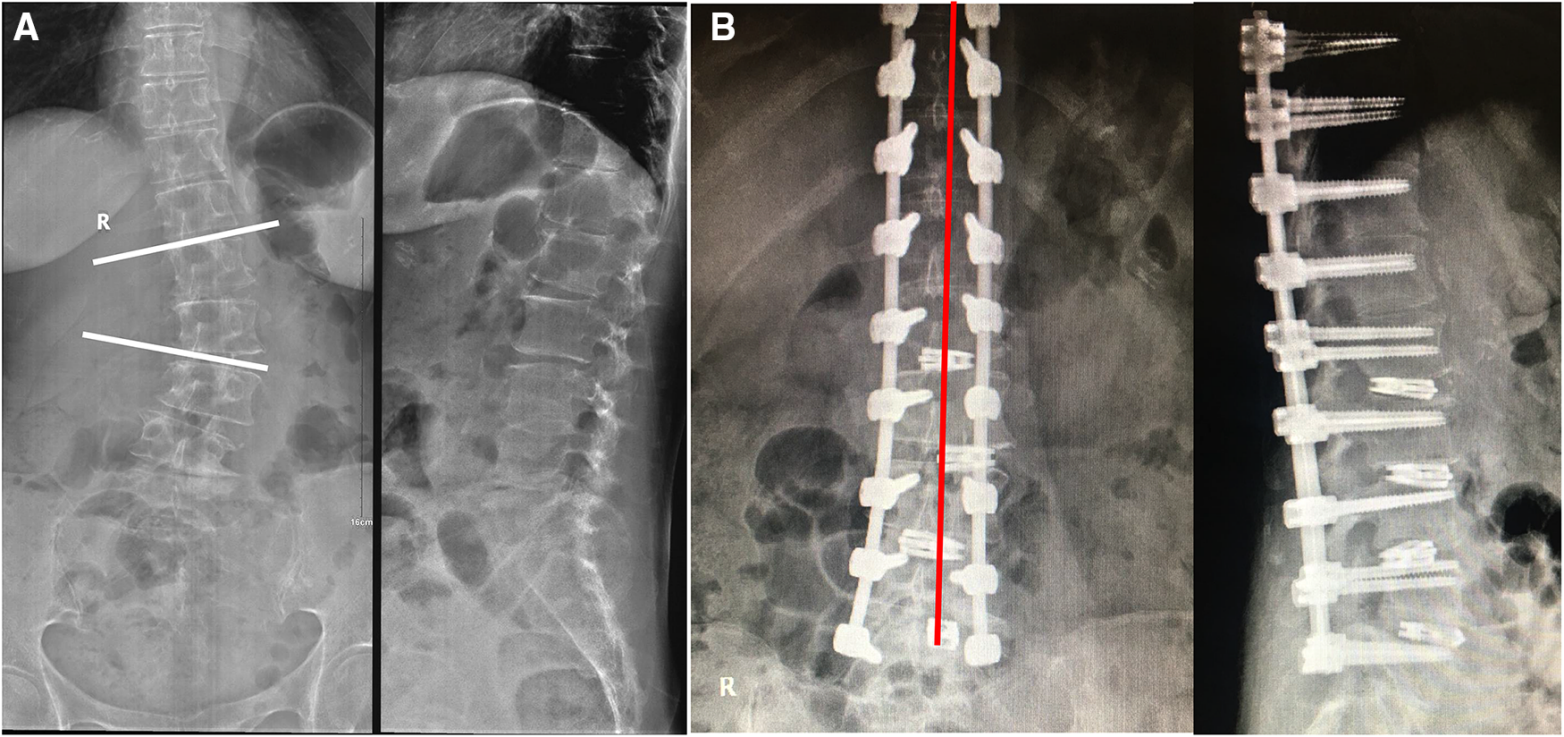
X-rays of the 61-year-old female patient with ADS, which was taken on December 2018: (**A**) before operation and (**B**) after operation.

A 61-year-old female presented with back pain and left lower leg pain for 20 years with progressive aggravation for more than 2 months. There were no cardiopulmonary deficits and no other underlying diseases. The muscle power of the hip as well as that of both lower limbs was 4/5 level upon physical examination, which was indicative of establishing a diagnosis of ADS. The VAS was 6 points for back pain and 5 points for leg pain, the ODI score was 64%, and the Silva–Lenke type was IV. Radiological examination showed lumbar scoliosis with a Cobb angle of 30 and LK with lordosis of 12° (Figure 2). During the past year, pain was severe, and was managed with traditional Chinese acupuncture and massage. However, no improvement was noted.

## Methods

In this report of two cases, we conducted all surgical procedures in accordance with the CARE guidelines (6). An informed consent was taken from both cases prior to examination or surgical correction. After both patients were indicated to have the surgery, our team conducted the necessary examinations and investigations preoperatively before correcting the scoliotic curve to adequately plan the surgical technique and have enhanced outcomes. The surgical approach was divided into two major steps. The first step was to perform intervertebral decompression and fusion with iliac bone grafting assisted by spinal endoscopy under intravenous anesthesia. The second step included the use of percutaneous pedicle screws for internal fixation and deformity correction under general anesthesia. Preoperative assessment, intraoperative manipulation, and postoperative appearance of both cases are provided (Figures 1, 2). The information of both cases was kept confidential by not providing any identifying data.

## Postoperative assessment

The deformity of the lumbar spine was corrected, and low back and leg pain were significantly relieved. No complications were observed during the follow-up period (Table 1). Pre- and postoperative data for both cases are reported in Table 1.

**Table 1:** General data and operation-related indexes of two patients.

Characteristic	Case 1	Case 2
Follow-up time (months)	24.3	25.5
Operation time (min)	144.5	154.6
Intraoperative blood loss (ml)	312.4	346.5
Back VAS (scores)		
Preoperative	9.0	6.0
Last follow-up	2.6	1.7
Leg VAS (scores)		
Preoperative	6.0	5.0
Last follow-up	2.7	1.5
ODI (%)		
Preoperative	76.0	64.0
Last follow-up	27.0	25.0
Cobb's angle (°)		
Preoperative	30.0	30.0
Last follow-up	15.7	16.6
SVA (mm)		
Preoperative	29.3	28.8
Last follow-up	28.1	28.0
LK (°)		
Preoperative	16.0	12.0
Last follow-up	14.5	10.7

VAS, visual analog scale; ODI, Oswestry disability index; SVA, sagittal vertical axis; LK, lumbar kyphosis.

## Discussion

Most surgical approaches proposed in the literature for the management of ADS aim to correct the resulting misalignment and neurogenic claudication, as well as to relieve the occurring back and lower limb pain. Treatment of ADS with percutaneous spinal endoscopic-assisted lumbar interbody fusion (EALIF) and percutaneous pedicle screw fixation has incomparable advantages (7–9). For instance, the percutaneous pedicle screw technique may avoid the sacrospinous muscle dissection, effectively reduce or avoid the spinal nerve dorsal branch injury, and protect the function of multiplex muscle. Over oblique lumbar interbody fusion (OLIF) is one type of lateral lumbar interbody fusion (LLIF), and through it, surgeons can access the disc space *via* an anterolateral approach between the aorta and psoas muscle. Direct lateral interbody fusion (DLIF) mitigates many of the vascular complications and bony resections associated with other interbody fusion techniques. However, there are concerns regarding postoperative neural complications since indirect decompression of the foramen has not been consistently demonstrated.

Recently, Heo et al. (10) assessed the efficacy of ALIF combined with percutaneous pedicle screw fixation in patients with adult lumbar spinal deformity. In their study, the ALIF technique was associated with favorable clinical and radiological outcomes. However, it is important to mention that the effects of ALIF were more evident in patients with sagittal vertical access >95 mm or pelvic tilt >30°. This finding is of critical importance in practice for proper patient selection and management. Furthermore, this technique is associated with non-negligible rates of vascular (up to 24%) and visceral injuries (up to 6%) (11–14). Therefore, the advent of another technique that provides preferable outcomes with reduced complications was deemed necessary. DLIF approach provides better outcomes compared to the previously mentioned approach in terms of reducing morbidity and complication rates (15, 16). First, this technique performs decompression under endoscopy, which is more advantageous than indirect decompression by DLIF and OLIF. Second, compared with the lateral anterior fusion of large vessels and abdominal organs, endoscopic fusion is less likely to damage nearby structures. Third, the endoscopic fusion surgery is performed under local anesthesia, and real-time feedback of patients reduces the risk of nerve structural damage. In addition, different MIS surgeries are selected for combined application according to different lesions in patients with ADS.

There are various advantages for conducting the surgical technique presented in this study. First, this technique performs decompression under endoscopy, which is more advantageous than indirect decompression by DLIF and OLIF. Second, compared with the lateral anterior fusion of large vessels and abdominal organs, endoscopic fusion is less likely to damage nearby structures. Third, the endoscopic fusion surgery is performed under local anesthesia, and real-time feedback of patients reduces the risk of nerve structural damage. However, our findings should be carefully interpreted due to several limitations. First, this article presents the findings of two cases only and when retrieving their relevant information, some were missed. Moreover, conducting adequate investigations was not feasible at our center since the necessary equipment was not available when the cases presented. Second, the follow-up period is not sufficient to provide a comprehensive conclusion about the long-term outcomes of this procedure. Therefore, prospective studies of larger sample sizes and longer follow-up periods are warranted to confirm the therapeutic potential of percutaneous spinal endoscopic-assisted lumbar interbody fusion with percutaneous pedicle screw fixation in patients with ADS. Additionally, we are not confident whether the reported outcomes are correlated with a degree of misalignment. Thus, we recommend future studies to include patients with variable clinical characteristics to determine if the effect of the reported technique would be modified based on these characteristics.

## Data Availability

The original contributions presented in the study are included in the article/Supplementary Material, further inquiries can be directed to the corresponding author.
